# Characterization of a Novel PolyM-Preferred Alginate Lyase from Marine *Vibrio splendidus* OU02

**DOI:** 10.3390/md16090295

**Published:** 2018-08-22

**Authors:** Jingjing Zhuang, Keke Zhang, Xiaohua Liu, Weizhi Liu, Qianqian Lyu, Aiguo Ji

**Affiliations:** 1 Marine College, Shandong University, Weihai 264209, China; zhuangjj929@sdu.edu.cn; 2 College of Marine Life Sciences, Ocean University of China, Qingdao 266003, China; airkele@sina.com (K.Z.); lingyun-lxh@163.com (X.L.); liuweizhi@ouc.edu.cn (W.L.); 3 School of Pharmaceutical Sciences, Shandong University, Jinan 250012, China

**Keywords:** alginate lyase, polysaccharide lyase family 7, *Vibrio splendidus* OU02, polyM-preferred, characterization

## Abstract

Alginate lyases are enzymes that degrade alginate into oligosaccharides which possess a variety of biological activities. Discovering and characterizing novel alginate lyases has great significance for industrial and medical applications. In this study, we reported a novel alginate lyase, AlyA-OU02, derived from the marine *Vibrio splendidus* OU02. The BLASTP searches showed that AlyA-OU02 belonged to polysaccharide lyase family 7 (PL7) and contained two consecutive PL7 domains, which was rare among the alginate lyases in PL7 family. Both the two domains, AlyA^a^ and AlyA^b^, had lyase activities, while AlyA^a^ exhibited polyM preference, and AlyA^b^ was polyG-preferred. In addition, the enzyme activity of AlyA^a^ was much higher than AlyA^b^ at 25 °C. The full-length enzyme of AlyA-OU02 showed polyM preference, which was the same as AlyA^a^. AlyA^a^ degraded alginate into di-, tri-, and tetra-alginate oligosaccharides, while AlyA^b^ degraded alginate into tri-, tetra-, and penta-alginate oligosaccharides. The degraded products of AlyA-OU02 were similar to AlyA^a^. Our work provided a potential candidate in the application of alginate oligosaccharide production and the characterization of the two domains might provide insights into the use of alginate of this organism.

## 1. Introduction

Alginate is a linear polysaccharide consisting of β-D-mannuronate (M) and α-L-guluronate (G) residues, arranged in varying sequences of poly β-D-mannuronate (polyM), poly α-L-guluronate (polyG), and the heteropolymer (polyMG) [1]. It has been widely used in biomedical science and engineering, due to its low toxicity, good biocompatibility, low cost, and mild gelation [2]. Alginate now has attracted increasing attention, since it is the most abundant polysaccharide of brown algae, which is considered as the third-generation biofuel [3]. Moreover, studies have showed that alginate oligosaccharides (AOS) possess a variety of biological activities, such as antioxidant, neuroprotective, antibacterial, and antitumor [4,5,6,7]. Therefore, to research the depolymerization of alginate has great importance.

Alginate lyases are enzymes that degrade alginate into unsaturated oligosaccharides by cleaving glycosidic bonds via a β-elimination mechanism [8]. To date, a number of alginate lyases have been identified from various organisms (e.g., algae, marine mollusks, bacteria, virus, and fungi) [2,8,9] and part of their structural relationships have been elucidated [10,11,12,13]. Alginate lyases are divided into polyM-specific, polyG-specific, and polyMG lyases, according to their substrate specificity [14]. According to the primary structures, alginate lyases are classified into seven polysaccharide lyase (PL) families, namely PL-5,6,7,14,15,17, and 18 [15]. Among the seven PL families, PL7 is the most prevalent as reported in the Carbohydrate-Active enZYmes database (http://www.cazy.org/). Most of the PL7 alginate lyases belong to polyG-specific lyases, and few belong to polyM-specific lyases [12]. The substrate specificity is related with the amino acids in the highly conserved regions [16,17]. Commonly, the polyM-specific, polyG-specific, and polyMG alginate lyases contain QVH, QIH, and QIH in the conserved regions, respectively [17].

**Figure 1:**
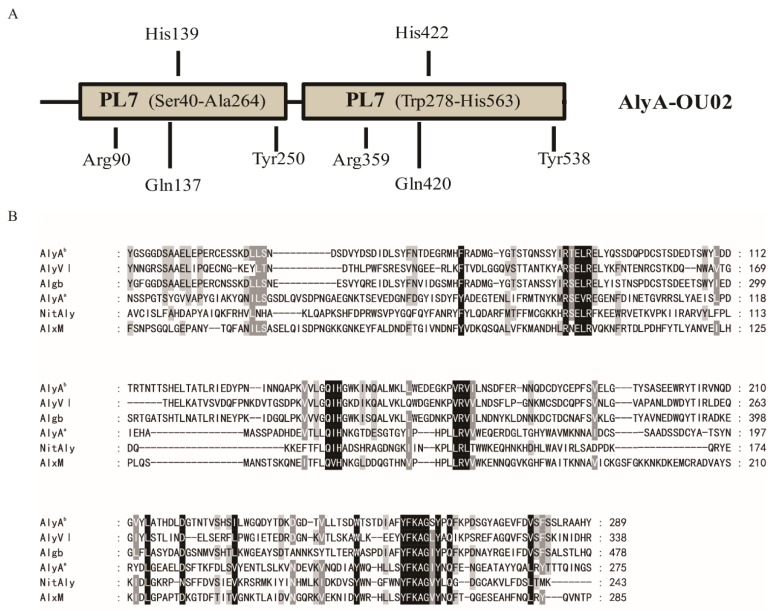
Sequence properties of the alginate lyase AlyA-OU02 from marine *Vibrio splendidus* OU02. (**A**) Module organization of AlyA-OU02. The two alginate_lyase 2 modules were putative catalytic domains (Ser^40^ to Ala^264^, Trp^278^ to His^563^). The full-length protein and the two alginate_lyase 2 modules were expressed to yield the recombinant protein AlyA-OU02 (Ser^1^ to Tyr^564^), AlyA^a^ (Ser^1^ to Ser^275^), and AlyA^b^ (Asn^276^ to Tyr^564^). The indicated amino acid residues were hypothesized catalytic sites. (**B**) Comparison of the partial amino acid sequences of AlyA^a^ and AlyA^b^ with PL7 alginate lyases AlyVI from *Vibrio* sp. QY101 (AAP45155.1), Algb from *Vibrio* sp. W13 (AIY22661.1), NitAly from *Nitratiruptor* sp. SB155-2 (BAF69299.1), AlxM from *Photobacterium* sp. ATCC 43367 (CAA49630.1).

In this study, we reported a new polyM-preferred alginate lyase (AlyA-OU02) from the marine *Vibrio splendidus OU02* which possessed two consecutive PL7 domains (Figure 1A). We cloned, expressed, and characterized AlyA-OU02 and the two PL7 domains (AlyA^a^ and AlyA^b^), respectively. Both PL7 domains contained a highly conserved QIH sequence which was thought to indicate ployG specificity. Interestingly, it turned out that AlyA-OU02 was polyM-preferred in accordance with AlyA^a^. Our work suggested the potential applications in AOS production of AlyA-OU02, and provided insights into the use of alginate of this organism.

## 2. Results and Discussions

### 2.1. Sequence Analysis of AlyA-OU02

The alginate lyase gene was cloned from marine *Vibrio splendidus* OU02, and was designated as AlyA-OU02. It consists of 1695 bp encoding 564 amino acids. The calculated molecular mass was ~64 kDa, and the isoelectric point (PI) was 4.27. The BLASTP searches showed that AlyA-OU02 contained two alginate_lyase2 superfamily modules, and belonged to PL7 family (Figure 1A), which was similar to AlyA from *Vibrio splendidus* 12B01 [18]. Figure 1B showed the multiple sequence alignments of AlyA^a^, AlyA^b^, and related alginate lyases of PL7 family [19,20,21,22]. Both domains contained the highly conserved regions R(S/T)E(V/L)R, QIH, and YFKAG(I/S)Y, which were necessary for substrate recognition and catalysis [23]. The putative residues involved in the catalytic reaction were pointed out in Figure 1A.

### 2.2. Cloning, Expression, and Purification of Different Versions of AlyA-OU02

**Figure 2:**
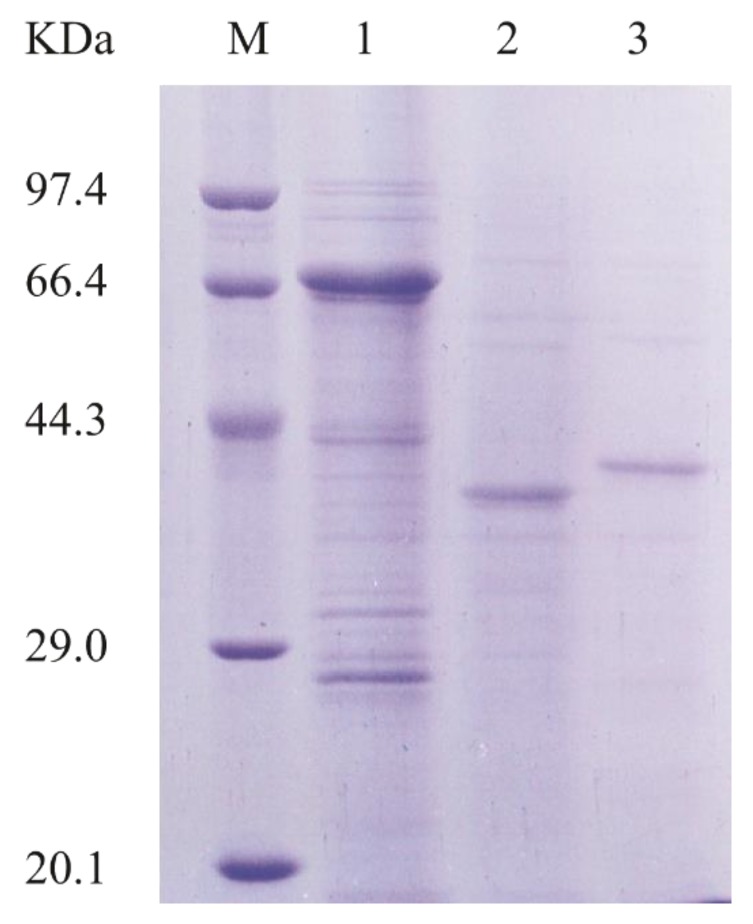
SDS-PAGE analysis of purified AlyA-OU02, AlyA^a^, and AlyA^b^. Lane M, molecular weight markers; Lane 1, purified AlyA-OU02; Lane 2, purified AlyA^a^; Lane 3, purified AlyA^b^.

To better characterize AlyA-OU02, we cloned, expressed, and purified AlyA-OU02 and its two domains, respectively. The AlyA-OU02, AlyA^a^, and AlyA^b^ genes were cloned into pET-32a-PreScission vector (modified by our laboratory) respectively, and expressed in the *E. coli* BL21 (DE3)-pET-32a-PreScission system. The enzymes were then purified by Ni-nitrilotriacetic acid agarose (Ni-NTA) affinity chromatography and analyzed by sodium dodecyl sulfate polyacrylamide gel electrophoresis (SDS-PAGE). Figure 2 showed that the molecular weights of AlyA-OU02, AlyA^a^, and AlyA^b^ were approximately 65 kDa, 31 kDa, and 33 kDa, which were consistent with the calculated molecular weights.

### 2.3. Enzymatic Activity Measurements for AlyA-OU02, AlyA^a^, and AlyA^b^

**Figure 3:**
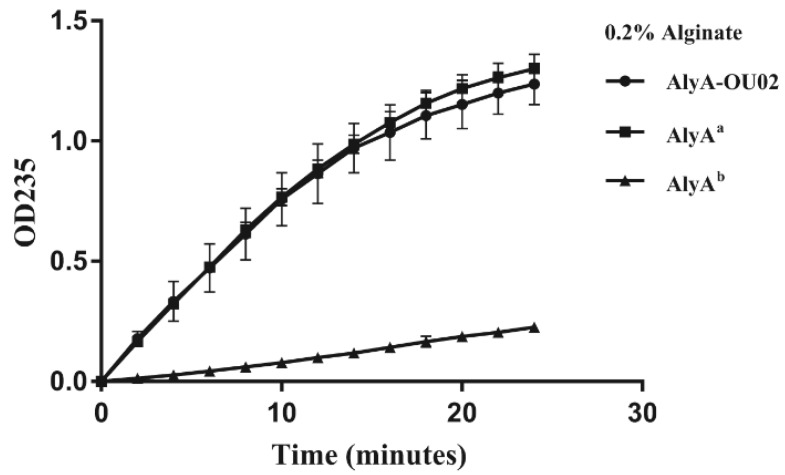
Enzyme activity assay of AlyA-OU02, AlyA^a^ and AlyA^b^ toward alginate. The measurement was carried out in Tris-HCl buffer with 200 mM NaCl (pH 7.5) using 0.2% alginate as substrate.

Alginate lyases could degrade alginate into unsaturated oligosaccharides with the formation of a double bond between C_4_ and C_5_ at the non-reducing terminus. The enzyme activity was measured by monitoring the increased absorbance at 235 nm. Both domains showed alginate lyase activities, and the enzyme activity of AlyA^a^ was much higher than AlyA^b^ at 25 °C (Figure 3). The enzyme activity of AlyA-OU02 was similar to AlyA^a^. The *k_m_* values of AlyA-OU02, AlyA^a^, and AlyA^b^ toward alginate were 3.55, 4.80, and 0.34 mg/mL respectively. The *k_cat_* values of AlyA-OU02, AlyA^a^, and AlyA^b^ toward alginate were 630, 1052, and 42 min^−1^, respectively. The enzymatic kinetics assay was consistent with the enzyme activity assay.

AlyA from *Vibrio splendidus* 12B01 also contained two PL7 domains, and the sequence similarity between AlyA-OU02 and AlyA was 89%, however, domain 1 of AlyA had no lyase activity [18]. We speculated the potential reason was that AlyA was purified by renaturation from inclusion bodies, and the length of AlyA (53–286) was different from AlyA^a^ [18]. 

Crystal structure of FlAlyA showed that His124 and Tyr239 in the highly conserved regions participated in the catalytic reaction [12]. Furthermore, the catalytic activities of the H124N/Y239F mutants were much lower than the wild type. We constructed H139N/Y250F mutants of AlyA^a^ and measured the lyase activities in order to identify the catalytic residues. The purified H139N/Y250F of AlyA^a^ were analyzed by SDS-PAGE (Figure S1). The alginate lyase activities of the mutants were undetectable, suggesting that His139 and Tyr250 were necessary for catalytic reaction of AlyA^a^ (Figure S2).

**Figure 4:**
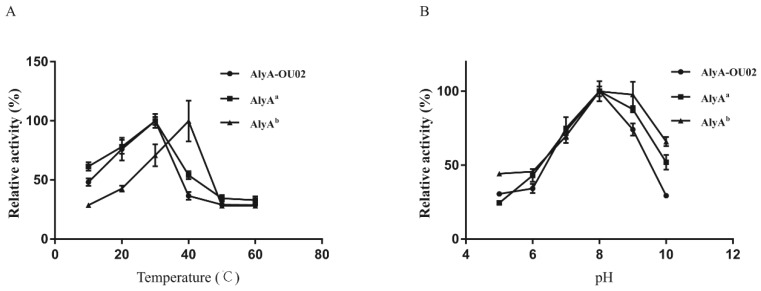
Effects of temperature and pH on enzyme activities of AlyA-OU02, AlyA^a^, and AlyA^b^. (**A**) Optimal temperatures of the enzymes were determined in Tris-HCl buffer with 200 mM NaCl (pH 7.5) at different temperatures. (**B**) Optimal pH of the enzymes was determined at 25 °C in NaAc-HAc buffer (pH 5–6) and Tris-HCl buffer (pH 7–10).

Alginate lyases with different sequences exhibited different optimal temperatures. Most of the PL7 alginate lyases in Table 1 had maximum activity at 30–45 °C. AlyA^a^ had maximum activity at 30 °C, while AlyA^b^ had maximum activity at 40 °C (Figure 4A). The different optimal temperature of the two domains might help the bacteria to better adapt to the changing environment. The alginate lyases in Table 1 had maximum activity in a pH range of 6.0 to 9.0. Both AlyA^a^ and AlyA^b^ exhibited maximum activity at pH 8.0, which corresponded with the range (Figure 4B). The optimal temperature/pH for AlyA-OU02 was 30 °C/8.0, the same as for AlyA^a^. Moreover, the optimal temperature/pH for Algb [20] and ALG-5 [24] in Table 1 was also 30 °C/8.0. Considering that the ion types in the buffer might impact the enzyme activity, we also investigated the pH effect in sodium phosphate buffer (pH 6.0–8.0) and Tris-HCl buffer (pH 7.0–10.0). Our results showed that sodium phosphate buffer decreased enzyme activities of AlyA-OU02, AlyA^a^, and AlyA^b^, compared with Tris-HCl buffer (Figure S3).

**Figure 5:**
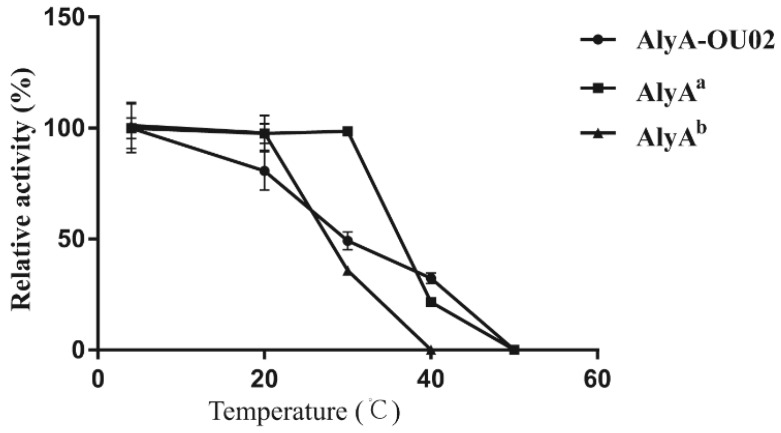
Thermostabilities of AlyA-OU02, AlyA^a^, and AlyA^b^.

To assess the thermostabilities of AlyA-OU02 and its two domains, the enzymes were incubated in Tris-HCl buffer at different temperatures for 1 h. The enzyme activities were assayed using alginate as the substrate. As shown in Figure 5, the activity of AlyA^a^ remained unchanged, while AlyA^b^ retained 40% activity after incubation at 30 °C for 1 h. The stability of AlyA-OU02 decreased with increasing temperature, and retained 50% activity after incubation at 30 °C for 1 h. All the three enzymes were inactive after incubation at 50 °C. Our findings suggested that AlyA^a^ exhibited more thermostability compared to AlyA^b^ and AlyA-OU02.

**Figure 6:**
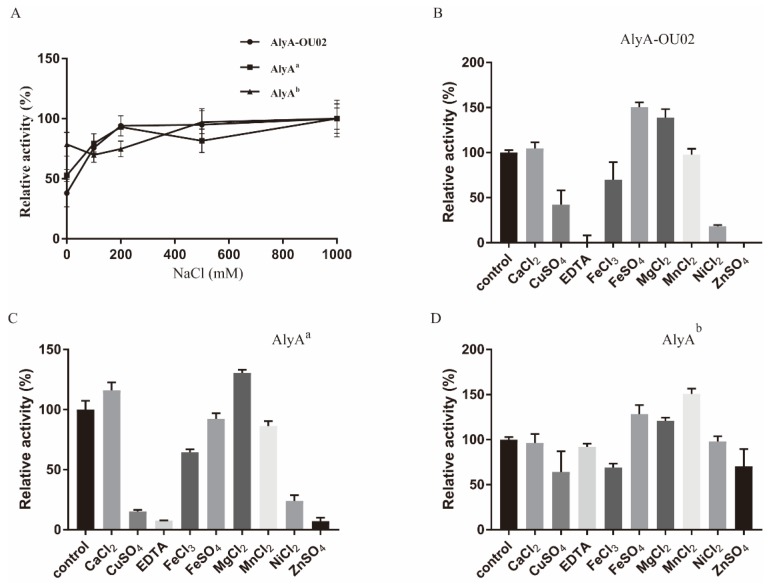
Effects of NaCl and metal ions on enzymatic activities of AlyA-OU02, AlyA^a^, and AlyA^b^. (**A**) Influence of the concentration of NaCl. (**B**) Influence of the metal ions on AlyA-OU02. (**C**) Influence of the metal ions on AlyA^a^. (**D**) Influence of the metal ions on AlyA^b^. The enzymatic activity without metal ions served as the control, and the enzymatic activity was designated as 100%.

The activities of alginate lyases might be affected by metal ions, since various organisms survived and evolved in various environments. We first examined the effects of NaCl on the enzymatic activities. As shown in Figure 6A, 200 mM NaCl could effectively enhance the activities of AlyA-OU02 and AlyA^a^, while moderately affecting AlyA^b^. Certain alginate lyases could be activated by NaCl, but were not resistant to high concentrations of NaCl, such as AlyH1 [36] and AlgA [39]. AlyA-OU02, AlyA^a^, and AlyA^b^ all exhibited high salt tolerance, which was analogous to AlgM4 from marine bacterium *Vibrio weizhoudaoensis* M0101 [32]. The effects of chelating agent and other metal ions on AlyA-OU02, AlyA^a^, and AlyA^b^ activities were studied in the absence of NaCl. As shown in Figure 6B–D, Ca^2+^ showed little effect on the enzymatic activities. Similarly to rAlgSV1-PL7 [35], AlyA-OU02, AlyA^a^, and AlyA^b^ could be activated by Mg^2+^. Besides, enzymatic activities of AlyA-OU02 and AlyA^a^ were inhibited by the chelating agent ethylenediaminetetraacetic acid (EDTA), indicating that AlyA-OU02 and AlyA^a^ might be ion-dependent alginate lyases. 

**Figure 7:**
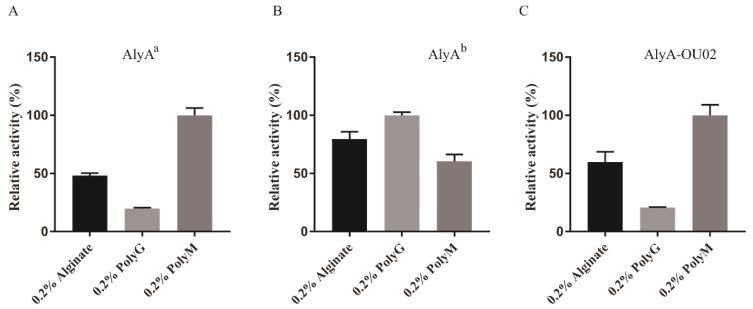
Substrate specificities of AlyA-OU02, AlyA^a^, and AlyA^b^. (**A**) Relative enzyme activities of AlyA^a^ toward alginate, polyM, and polyG. (**B**) Relative enzyme activities of AlyA^b^ toward alginate, polyM, and polyG. (**C**) Relative enzyme activities of AlyA-OU02 toward alginate, polyM, and polyG.

To determine the substrate specificity, 0.2% alginate, polyG, and polyM were used as the substrates to study the enzyme activities. The relative activities of AlyA^a^ toward alginate, polyG, and polyM were 48.1% ± 2.2%, 20.0% ± 2.2%, and 100.0% ± 6.2%, respectively, indicating that AlyA^a^ was a polyM-preferred alginate lyase (Figure 7A). AlyA^b^ showed slight polyG preference (Figure 7B). The relative activities of AlyA-OU02 toward alginate, polyG, and polyM, were 60.0% ± 8.7%, 20.7% ± 0.4%, and 100.0% ± 9.1%, respectively (Figure 7C), which was similar to AlyA^a^. The specific activities of AlyA-OU02, AlyA^a^, and AlyA^b^ toward alginate, polyG, and polyM are shown in Table 2. The substrate specificities of PL7 alginate lyases were related with the protein sequences in the conserved regions [17]. Crystal structures and sequence analyses showed that the three conserved regions in PL7 family formed the cavity composed of a jelly roll β-sandwich structure, which was assumed to bind to a suitable substrate [24]. The conserved amino acids are thought to be pivotal in catalytic activity or folding of the structure. Recent studies have revealed that the polyM-specific, polyG-specific, and polyMG alginate lyases contained QVH, QIH, and QIH in the conserved regions, respectively [17]. As shown in Table 1, the alginate lyases that showed activities toward polyG and polyM contained QIH in the conserved region, with the exception of rAlgSV1-PL7 [35]. A9mT and PyAly contained QVH and were polyM-specific [24,38]. The polyG-specific enzymes, such as Alg2A, ALG-5, and Aly2, all contained QIH sequence [26,30,37]. Contrary to this accepted rule, AlyA^a^ was active toward polyM, containing QIH sequence which was similar to FlAlyA and AlyDW11 [27,31].

### 2.4. Thin-Layer Chromatography Analysis of the Degradation Products

**Figure 8:**
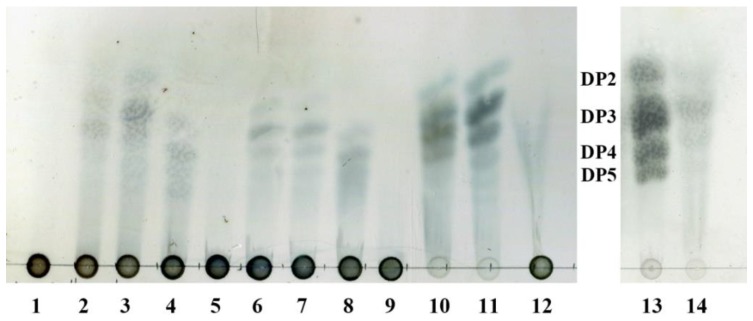
TLC analysis of the hydrolytic products of AlyA-OU02, AlyA^a^, and AlyA^b^. Solutions of 0.2% (*w*/*v*) alginate, polyG, and polyM were each incubated with 5 uM AlyA-OU02, AlyA^a^, and AlyA^b^, respectively, at 25 °C for 1 h. Lane 1: alginate; lane 2–4: hydrolytic products of AlyA-OU02, AlyA^a^, and AlyA^b^ toward alginate; lane 5: polyG; lane 6–8: hydrolytic products of AlyA-OU02, AlyA^a^, and AlyA^b^ toward polyG; lane 9: polyM; lane 10–12: hydrolytic products of AlyA-OU02, AlyA^a^, and AlyA^b^ toward polyM; lane 13: DP2, DP3, DP4, and DP5 mean the alginate disaccharide, trisaccharide, tetrasaccharide, and pentasaccharide, respectively; lane 14: hydrolytic products of AlyA^a^ toward polyM.

To investigate the action modes of AlyA-OU02, AlyA^a^, and AlyA^b^, the degradation products of alginate, polyG, and polyM after 1 h were analyzed by thin-layer chromatography (TLC). As shown in Figure 8, AlyA^a^ degraded alginate and polyM into di-, tri-, and tetra-oligosaccharides, and trisaccharide was the main product. The products patterns of AlyA-OU02 were similar to AlyA^a^. For AlyA^b^, tri-, tetra-, and penta-oligosaccharides were the main hydrolysis products, and AlyA^b^ had limited activity toward polyM. The results were consistent with the enzymatic assays. We also analyzed the overnight products of alginate, polyG, and polyM, and the product patterns remained the same (Figure S4). This indicated that AlyA-OU02 and AlyA^a^ might be potential tools for preparation of lower molecular weight polyM products which have wide pharmaceutical applications.

## 3. Materials and Methods 

### 3.1. Materials

Alginate was purchased from Shanghai Yuanye co., Ltd. The alginate viscosity is 4500 cP at 2% (*w*/*v*) concentration at 25 °C. The M/G ratio of alginate is 0.6 as determined by NMR analysis. PolyG and polyM were prepared according to the reference [10]. Other chemicals and reagents used in this study were of analytical grade.

### 3.2. Sequence Analysis of AlyA-OU02

For functional annotation, the BLAST algorithm on the National Center for Biotechnology Information server (http://www.ncbi.nlm.nih.gov) was used to analyze the similarity of the amino acid sequences. Molecular weights of the enzymes were estimated by the peptide mass tool on the ExPASY server of the Swiss Institute of Bioinformatics (http://swissmodel.expasy.org/). Multiple sequence alignments were performed by MEGA version 5.05.

### 3.3. Construction of Expression Vectors

The gene encoding AlyA-OU02 was amplified from genomic *Vibrio splendidus* OU02 DNA (unpublished data) using the forward primer (5’-CGGAATTCAGTAGTTCAAATAGCTCGACTG-3′) and reverse primer (5′-CCGCTCGAGTCAATAGTGTGCCGCTCTAAGAG-3′). The AlyA^a^ gene was amplified using the forward primer (5’-GGAATTCAGTAGTTCAAATAGCTCGACTG-3′) and reverse primer (5′-CCGCTCGAGTTATGACCCATTGATTTG-3′). The AlyA^b^ gene was amplified using the forward primer (5’-GGAATTCAACGATTGGGACATTAATGATTG-3′) and reverse primer (5′-CCGCTCGAGTTAATAGTGTGCCGCTCTAAG-3′). DNA amplification products were cloned into the expression vector pET-32a-PreScission. The recombinant plasmids were transformed into *E. coli* BL21 (DE3) cells. DNA sequencing was used to confirm the integrities of the nucleotide sequences of newly constructed plasmids. 

Site-directed mutagenesis was conducted using the QuikChange Site-Directed Mutagenesis Kit (Aglient Technologies, CA, USA). For generation of the H139N mutant, the primers used in this study were forward primer (5′-GTGACGTTGCTGCAGATAAACAATAAGGGGACTGATG-3′) and reverse primer (5′-CATCAGTCCCCTTATTGTTTATCTGCAGCAACGTCAC-3′). For the Y250F mutant, the primers used in this study were forward primer (5′-GCTACTTCAAAGCGGGTATCTTTAACCA``ATTTGAGAATGGTG-3′) and reverse primer (5′-CACCATTCTCAAATTGGTTAAAGATACCCGCTTTGAAGTAGC-3′). 

### 3.4. Expression and Purification of AlyA-OU02, AlyA^a^ and AlyA^b^

*E. coli* BL21 (DE3) cells harboring the plasmid were initially cultured in LB broth containing ampicillin at 37 °C. When the cell density reached an A_600_ value of 0.6, 0.1 mM (final concentration) isopropyl β-d-1-thiogalactopyranoside was used to induce enzyme expression for 8 h at 16 °C. The cells were harvested by centrifugation (1500× *g*, 30 min, 4 °C) and sonicated in 20 mM Tris, 500 mM NaCl (pH 7.5). The lysates were clarified by centrifugation (20,000× *g*, 30 min, 4 °C). The supernatant containing soluble proteins was loaded onto a Ni-NTA column. The samples were eluted with a linear gradient of 10–500 mM imidazole. The molecular mass and the purities of the enzymes were analyzed by 12% SDS-PAGE. 

### 3.5. Enzymatic Activity Assay

To determine the activities of the alginate lyases, 20 μL diluted enzyme (5 μM) was added to 180 μL substrate solution containing 0.2% (*w*/*v*) substrate, 50 mM Tris-HCl, and 200 mM NaCl (pH 7.5). After incubation at 25 °C for 10 min, alginate lyase activity was determined by measuring the increased absorbance at 235 nm of the reaction buffer. One unit was defined as the amount of enzyme required to increase the absorbance at 235 nm by 0.1 per min [40].

For kinetic analysis, the substrate concentrations varied from 0.33 mg/mL to 4 mg/mL. The reactions (200 μL for each assay) were carried out in 50 mM Tris-HCl (pH 7.5) with 200 mM NaCl at 25 °C for 10 min. After incubation, 200 μL of dinitrosalicylic acid was added to the reaction mixture, followed by heating at 100 °C for 5 min and centrifugation. The absorbance at 520 nm of the resulting supernatant was measured. 

To determine the effects of temperature on enzyme activities, the reactions (200 μL for each assay) were carried out in 50 mM Tris-HCl (pH 7.5) with 200 mM NaCl at different temperatures (10–60 °C) for 10 min, with alginate as substrate. After incubation, 200 μL of dinitrosalicylic acid was added to the reaction mixture, followed by heating at 100 °C for 5 min and centrifugation. The absorbance at 520 nm of the resulting supernatant was measured. To determine the effects of pH on enzyme activities, the reactions (200 μL for each assay) were carried out in buffers with different pH values, including NaAC-HAC buffer (pH 5.0–6.0) and Tris-HCl buffer (pH 7.0–10.0) at 25 °C for 10 min, with alginate as substrate. The increased absorbance at 520 nm was measured.

To determine the thermostabilities, the enzyme solutions were incubated at various temperatures for 1 h. The reactions (200 μL for each assay) were carried out in 50 mM Tris-HCl (pH 7.5) with 200 mM NaCl at 25 °C for 10 min with 0.2% (*w*/*v*) alginate as substrate. After incubation, 200 μL of dinitrosalicylic acid was added to the reaction mixture, followed by heating at 100 °C for 5 min and centrifugation. The absorbance at 520 nm of the resulting supernatant was measured.

The effects of metal ions on enzyme activities were carried out by incubating enzymes at 4 °C for 1 h in the presence of various metal ions (1 mM) or EDTA (5 mM) in 50 mM Tris-HCl buffer (pH 7.5). The reactions (200 μL for each assay) were carried out in 50 mM Tris-HCl (pH 7.5) at 25 °C for 10 min with alginate as substrate. The increased absorbance at 235 nm was measured. 

To study the substrate specificities, the reactions (200 μL for each assay) were carried out in 50 mM Tris-HCl (pH 7.5) with 200 mM NaCl at 25 °C for 10 min, using 0.2% (*w*/*v*) alginate, polyM, and polyG separately as substrates. The increased absorbance at 235 nm was measured. 

### 3.6. TLC Analysis of the Degradation Products

To analyze the oligosaccharides produced by the enzymes, 20 μL diluted enzyme (5 μM) was added to 180 μL substrate solution containing 0.2% (*w*/*v*) substrate, 50 mM Tris-HCl, and 200 mM NaCl (pH 7.5). After incubation at 25 °C for 1 h or 12 h, the reaction buffer was boiled for 5 min, and analyzed by TLC with the solvent system (1-butanol/formic acid/water 4:6:1). The TLC plate was sprayed using 10% (*v*/*v*) sulfuric acid in ethanol, and then heated at 130 °C for 5 min. 

## 4. Conclusions

In this study, we reported a new alginate lyase derived from the marine *Vibrio splendidus* OU02. AlyA-OU02 was characterized as a novel PL7 alginate lyase containing two PL7 domains. Surprisingly, both of the two domains showed alginate lyase activities. Although both domains contained QIH sequence in the conserved regions, which was thought to be polyG-specific, AlyA-OU02 turned out to be polyM-preferred. The two domains exhibited different enzymatic properties. The optimal temperature, pH, metal ions effects, and degradation patterns of AlyA-OU02 were similar to AlyA^a^. The putative reason was that the enzyme activity of AlyA^a^ was much higher than AlyA^b^ at 25 °C. Further work will be focused on obtaining crystals of the enzymes to elucidate the catalytic mechanism of AlyA-OU02.

## Figures and Tables

**Table 1:** Characterization of alginate lyases in PL7 family.

Protein Name	Optimal pH/Temperature (°C)	Conserved Region QIH/QVH	Substrate Specificity	Reference
AlyL1	8.6/40	QIH	PM, PG	[25]
Alg2A	8.5/45	QIH	PG	[26]
FlAlyA	7.8/55	QIH	PM	[27]
AlyPI	7.0/40	QIH	PM, PG	[28]
AI-II′	7.5/40	QIH	PM, PG, PMG	[29]
ALG-5	8.0/30	QIH	PG	[30]
AlyDW11	7.0/45	QIH	PM	[31]
A9MT	7.5/30	QVH	PM	[24]
AlgM4	8.5/30	QIH	PM, PG	[32]
AlgNJU-03	7.0/30	QIH	PM, PG	[33]
AlgNJ-07	9.0/40	-	PM	[34]
rAlgSV1-PL7	8.0/45	QVH	PM, PG, PMG	[35]
AlyH1	7.5/40	QIH	PM, PG	[36]
Aly2	6.0/40	QIH	PG	[37]
Algb	8.0/30	QIH	PM, PG	[20]
PyAly	8.0/35	QVH	PM	[38]
AlyVI	7.5/40	QIH	PM, PG	[19]
NitAly	6.0/70	QIH	PM	[21]
AlxM	-/-	QVH	PM	[22]

**Table 2:** Specific activities of AlyA-OU02, AlyA^a^, and AlyA^b^ toward alginate, polyG, and polyM.

Specific Activity (U/mg)	Alginate	PolyG	PolyM
AlyA-OU02	119	41	198
AlyA^a^	252	104.5	520
AlyA^b^	24	33	15

## Supplementary Materials

The following are available online at http://www.mdpi.com/1660-3397/16/9/295/s1, Figure S1: SDS-PAGE analysis of purified H139N/Y250F mutants of AlyA^a^. Lane 1, H139 mutant of AlyA^a^; Lane 2, Y250F mutants of AlyA^a^; Lane M, molecular weight markers, Figure S2: Enzyme activities of H139 and Y250F mutants of AlyA^a^. The relative enzyme activity of AlyA^a^ was designated as 100%, Figure S3: Effects of pH on enzyme activities of AlyA-OU02, AlyA^a^, and AlyA^b^. pH effects on enzyme activities of AlyA-OU02 (**A**), AlyA^a^ (**B**), and AlyA^b^ (**C**) in sodium phosphate buffer (pH 6.0–8.0) and Tris-HCl buffer (pH 7.0–10.0), Figure S4: TLC analysis of the hydrolytic products of AlyA-OU02, AlyA^a^, and AlyA^b^. Analysis of the hydrolytic products of AlyA-OU02 (lane 1–3), AlyA^a^ (lane 4–6), and AlyA^b^ (lane 7–9) for 12 h with either alginate, polyG, and polyM as substrates. Lane 10, DP2, DP3, DP4, and DP5 mean the alginate disaccharide, trisaccharide, tetrasaccharide, and pentasaccharide, respectively.

## References

[1] Pawar S.N., Edgar K.J. (2012). Alginate derivatization: A review of chemistry, properties and applications. Biomaterials.

[2] Lee K.Y., Mooney D.J. (2012). Alginate: Properties and biomedical applications. Prog. Polym. Sci..

[3] Wargacki A.J., Leonard E., Win M.N., Regitsky D.D., Santos C.N., Kim P.B., Cooper S.R., Raisner R.M., Herman A., Sivitz A.B. (2012). An engineered microbial platform for direct biofuel production from brown macroalgae. Science.

[4] Falkeborg M., Cheong L.Z., Gianfico C., Sztukiel K.M., Kristensen K., Glasius M., Xu X., Guo Z. (2014). Alginate oligosaccharides: Enzymatic preparation and antioxidant property evaluation. Food Chem..

[5] Tusi S.K., Khalaj L., Ashabi G., Kiaei M., Khodagholi F. (2011). Alginate oligosaccharide protects against endoplasmic reticulum- and mitochondrial-mediated apoptotic cell death and oxidative stress. Biomaterials.

[6] An Q.D., Zhang G.L., Wu H.T., Zhang Z.C., Zheng G.S., Luan L., Murata Y., Li X. (2009). Alginate-deriving oligosaccharide production by alginase from newly isolated Flavobacterium sp. LXA and its potential application in protection against pathogens. J. Appl. Microbiol..

[7] Chen J., Hu Y., Zhang L., Wang Y., Wang S., Zhang Y., Guo H., Ji D., Wang Y. (2017). Alginate Oligosaccharide DP5 Exhibits Antitumor Effects in Osteosarcoma Patients following Surgery. Front. Pharmacol..

[8] Ertesvag H. (2015). Alginate-modifying enzymes: Biological roles and biotechnological uses. Front. Microbiol..

[9] Suzuki H., Suzuki K., Inoue A., Ojima T. (2006). A novel oligoalginate lyase from abalone, Haliotis discus hannai, that releases disaccharide from alginate polymer in an exolytic manner. Carbohydr. Res..

[10] Lyu Q., Zhang K., Zhu Q., Li Z., Liu Y., Fitzek E., Yohe T., Zhao L., Li W., Liu T. (2018). Structural and biochemical characterization of a multidomain alginate lyase reveals a novel role of CBM32 in CAZymes. Biochim. Biophys. Acta.

[11] Ogura K., Yamasaki M., Mikami B., Hashimoto W., Murata K. (2008). Substrate recognition by family 7 alginate lyase from *Sphingomonas* sp. A1. J. Mol. Biol..

[12] Qin H.M., Miyakawa T., Inoue A., Nishiyama R., Nakamura A., Asano A., Ojima T., Tanokura M. (2018). Structural basis for controlling the enzymatic properties of polymannuronate preferred alginate lyase FlAlyA from the PL-7 family. Chem. Commun..

[13] Matsubara Y., Kawada R., Iwasaki K., Kimura Y., Oda T., Muramatsu T. (2000). Cloning and sequence analysis of a gene (aly PG) encoding poly (alpha-L-guluronate) lyase from *Corynebacterium* sp. strain ALY-1. J. Biosci. Bioeng..

[14] Wong T.Y., Preston L.A., Schiller N.L. (2000). Alginate lyaseL: Review of major sources and enzyme characteristics, structure-function analysis, biological roles, and applications. Annu. Rev. Microbiol..

[15] Lombard V., Bernard T., Rancurel C., Brumer H., Coutinho P.M., Henrissat B. (2010). A hierarchical classification of polysaccharide lyases for glycogenomics. Biochem. J..

[16] Osawa T., Matsubara Y., Muramatsu T., Kimura M., Kakuta Y. (2005). Crystal structure of the alginate (poly alpha-l-guluronate) lyase from *Corynebacterium* sp. at 1.2 A resolution. J. Mol. Biol..

[17] Zhu B., Yin H. (2015). Alginate lyase: Review of major sources and classification, properties, structure-function analysis and applications. Bioengineered.

[18] Badur A.H., Jagtap S.S., Yalamanchili G., Lee J.K., Zhao H., Rao C.V. (2015). Alginate lyases from alginate-degrading Vibrio splendidus 12B01 are endolytic. Appl. Environ. Microbiol..

[19] Han F., Gong Q.H., Song K., Li J.B., Yu W.G. (2004). Cloning, sequence analysis and expression of gene alyVI encoding alginate lyase from marine bacterium Vibrio sp. QY101. DNA Seq..

[20] Zhu B., Tan H., Qin Y., Xu Q., Du Y., Yin H. (2015). Characterization of a new endo-type alginate lyase from Vibrio sp. W13. Int. J. Biol. Macromol..

[21] Inoue A., Anraku M., Nakagawa S., Ojima T. (2016). Discovery of a Novel Alginate Lyase from *Nitratiruptor* sp. SB155-2 Thriving at Deep-sea Hydrothermal Vents and Identification of the Residues Responsible for Its Heat Stability. J. Biol. Chem..

[22] Chavagnat F., Heyraud A., Colin-Morel P., Guinand M., Wallach J. (1998). Catalytic properties and specificity of a recombinant, overexpressed D-mannuronate lyase. Carbohydr. Res..

[23] Yamasaki M., Moriwaki S., Miyake O., Hashimoto W., Murata K., Mikami B. (2004). Structure and function of a hypothetical Pseudomonas aeruginosa protein PA1167 classified into family PL-7: A novel alginate lyase with a beta-sandwich fold. J. Biol. Chem..

[24] Uchimura K., Miyazaki M., Nogi Y., Kobayashi T., Horikoshi K. (2010). Cloning and sequencing of alginate lyase genes from deep-sea strains of Vibrio and Agarivorans and characterization of a new Vibrio enzyme. Mar. Biotechnol..

[25] Li S., Yang X., Zhang L., Yu W., Han F. (2015). Cloning, Expression, and Characterization of a Cold-Adapted and Surfactant-Stable Alginate Lyase from Marine Bacterium Agarivorans sp. L11. J. Microbiol. Biotechnol..

[26] Huang L., Zhou J., Li X., Peng Q., Lu H., Du Y. (2013). Characterization of a new alginate lyase from newly isolated Flavobacterium sp. S20. J. Ind. Microbiol. Biotechnol..

[27] Inoue A., Takadono K., Nishiyama R., Tajima K., Kobayashi T., Ojima T. (2014). Characterization of an alginate lyase, FlAlyA, from Flavobacterium sp. strain UMI-01 and its expression in Escherichia coli. Mar. Drugs.

[28] Duan G., Han F., Yu W. (2009). Cloning, sequence analysis, and expression of gene alyPI encoding an alginate lyase from marine bacterium Pseudoalteromonas sp. CY24. Can. J. Microbiol..

[29] Miyake O., Ochiai A., Hashimoto W., Murata K. (2004). Origin and diversity of alginate lyases of families PL-5 and -7 in Sphingomonas sp. strain A1. J. Bacteriol..

[30] Kim D.E., Lee E.Y., Kim H.S. (2009). Cloning and characterization of alginate lyase from a marine bacterium Streptomyces sp. ALG-5. Mar. Biotechnol..

[31] Sim S.J., Baik K.S., Park S.C., Choe H.N., Seong C.N., Shin T.S., Woo H.C., Cho J.Y., Kim D. (2012). Characterization of alginate lyase gene using a metagenomic library constructed from the gut microflora of abalone. J. Ind. Microbiol. Biotechnol..

[32] Huang G., Wang Q., Lu M., Xu C., Li F., Zhang R., Liao W., Huang S. (2018). AlgM4: A New Salt-Activated Alginate Lyase of the PL7 Family with Endolytic Activity. Mar. Drugs.

[33] Zhu B., Sun Y., Ni F., Ning L., Yao Z. (2018). Characterization of a new endo-type alginate lyase from *Vibrio* sp. NJU-03. Int. J. Biol. Macromol..

[34] Zhu B., Hu F., Yuan H., Sun Y., Yao Z. (2018). Biochemical Characterization and Degradation Pattern of a Unique pH-Stable PolyM-Specific Alginate Lyase from Newly Isolated Serratia marcescens NJ-07. Mar. Drugs.

[35] Yagi H., Isobe N., Itabashi N., Fujise A., Ohshiro T. (2017). Characterization of a Long-Lived Alginate Lyase Derived from Shewanella Species YH1. Mar. Drugs.

[36] Zhu X., Li X., Shi H., Zhou J., Tan Z., Yuan M., Yao P., Liu X. (2018). Characterization of a Novel Alginate Lyase from Marine Bacterium Vibrio furnissii H1. Mar. Drugs.

[37] Peng C., Wang Q., Lu D., Han W., Li F. (2018). A Novel Bifunctional Endolytic Alginate Lyase with Variable Alginate-Degrading Modes and Versatile Monosaccharide-Producing Properties. Front. Microbiol..

[38] Inoue A., Mashino C., Uji T., Saga N., Mikami K., Ojima T. (2015). Characterization of an Eukaryotic PL-7 Alginate Lyase in the Marine Red Alga Pyropia yezoensis. Curr. Biotechnol..

[39] Chen P., Zhu Y., Men Y., Zeng Y., Sun Y. (2018). Purification and Characterization of a Novel Alginate Lyase from the Marine Bacterium Bacillus sp. Alg07. Mar. Drugs.

[40] Han W., Gu J., Cheng Y., Liu H., Li Y., Li F. (2016). Novel Alginate Lyase (Aly5) from a Polysaccharide-Degrading Marine Bacterium, *Flammeovirga* sp. Strain MY04: Effects of Module Truncation on Biochemical Characteristics, Alginate Degradation Patterns, and Oligosaccharide-Yielding Properties. Appl. Environ. Microbiol..

