# Supplementary figures and images for: Characterization of a Novel PolyM-Preferred Alginate Lyase from Marine Vibrio splendidus OU02
Source: Mar Drugs. 2018 Aug 22;16(9):295. doi: 10.3390/md16090295 (PMC6165035; doi:10.3390/md16090295)

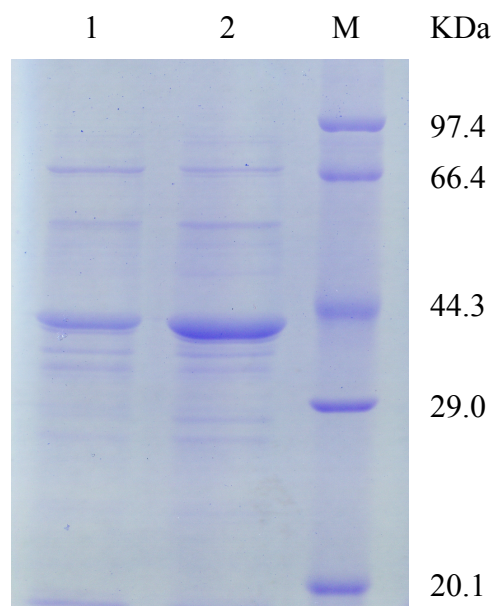

Supplement: Supplementary file 1 [file marinedrugs-16-00295-s001.zip › Supplementary figures/Figure S1.pdf]

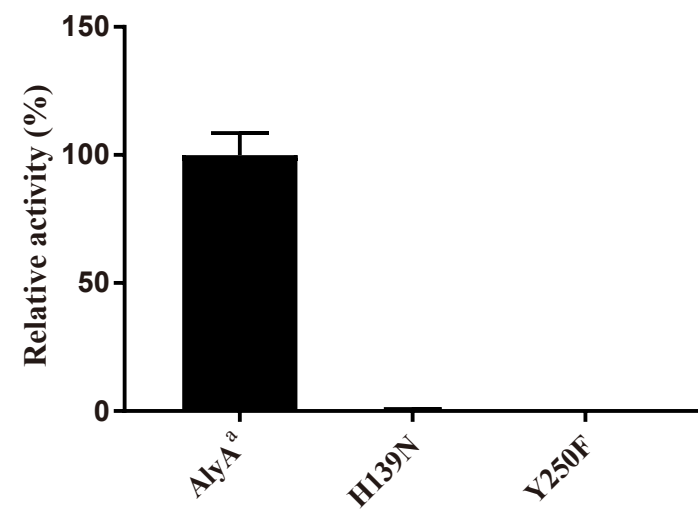

Supplement: Supplementary file 1 [file marinedrugs-16-00295-s001.zip › Supplementary figures/Figure S2.pdf]

**A**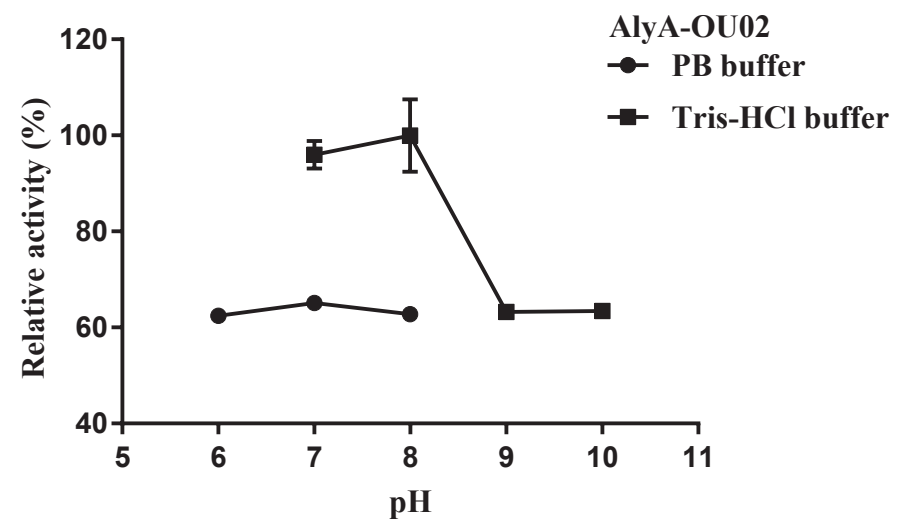**B**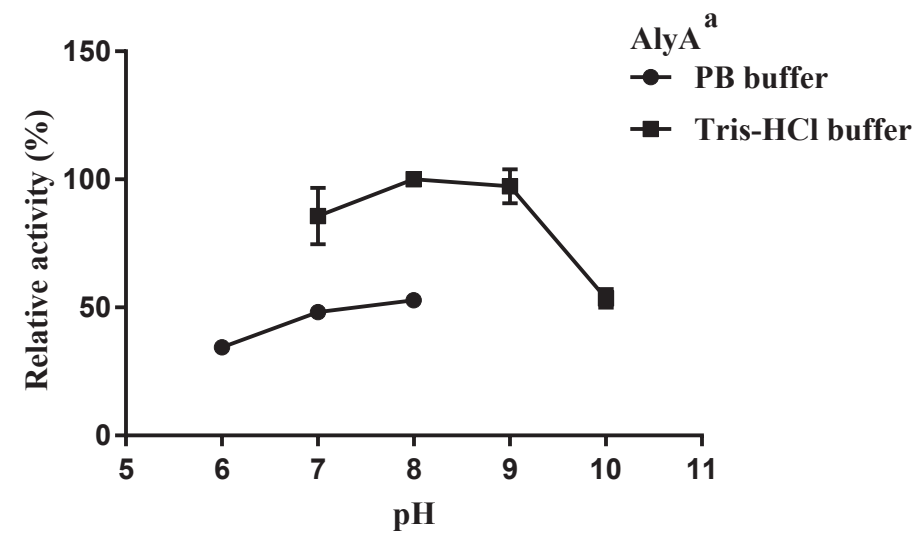**C**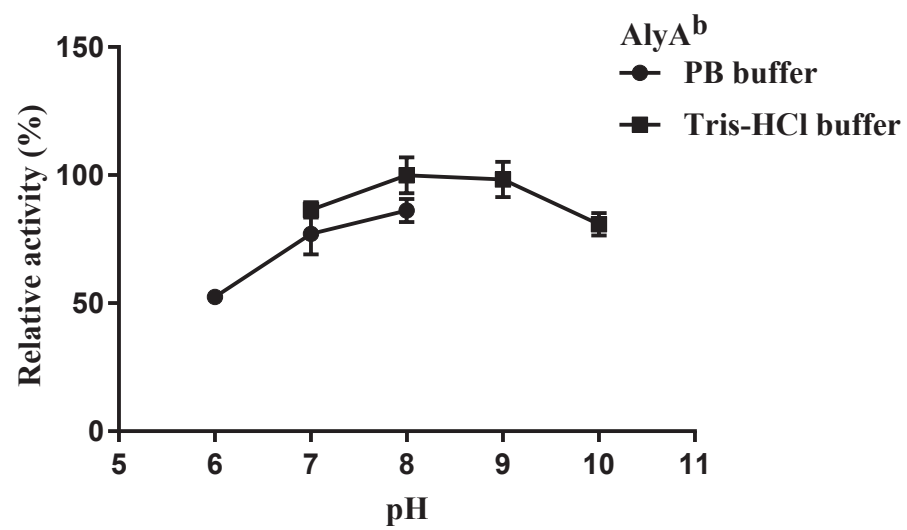

Supplement: Supplementary file 1 [file marinedrugs-16-00295-s001.zip › Supplementary figures/Figure S3.pdf]

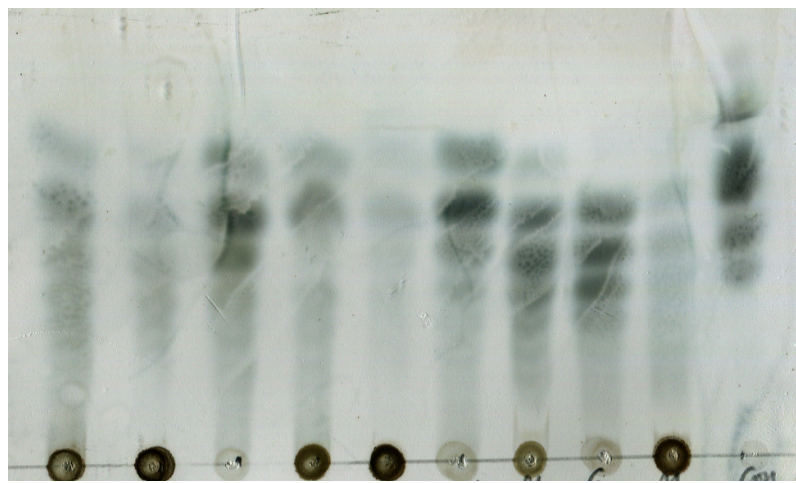

DP2

DP3

DP4

DP5

1

2

3

4

5

6

7

8

9

10

Supplement: Supplementary file 1 [file marinedrugs-16-00295-s001.zip › Supplementary figures/Figure S4.pdf]
